# PAM50 gene signatures and breast cancer prognosis with adjuvant anthracycline- and taxane-based chemotherapy: correlative analysis of C9741 (Alliance)

**DOI:** 10.1038/npjbcancer.2015.23

**Published:** 2016-01-06

**Authors:** Minetta C Liu, Brandelyn N Pitcher, Elaine R Mardis, Sherri R Davies, Paula N Friedman, Jacqueline E Snider, Tammi L Vickery, Jerry P Reed, Katherine DeSchryver, Baljit Singh, William J Gradishar, Edith A Perez, Silvana Martino, Marc L Citron, Larry Norton, Eric P Winer, Clifford A Hudis, Lisa A Carey, Philip S Bernard, Torsten O Nielsen, Charles M Perou, Matthew J Ellis, William T Barry

**Affiliations:** 1 Department of Oncology, Department of Laboratory Medicine and Pathology, Mayo Clinic, Rochester, MN, USA; 2 Department of Biostatistics and Bioinformatics, Alliance Statistics and Data Center, Duke University Medical Center, Durham, NC, USA; 3 The Genome Institute, Washington University, St. Louis, MO, USA; 4 Department of Medicine, Washington University School of Medicine, St. Louis, MO, USA; 5 Alliance for Clinical Trials in Oncology, University of Chicago, Chicago, IL, USA; 6 Department of Pathology, Washington University, St. Louis, MO, USA; 7 Department of Pathology, New York University Medical Center, New York, NY, USA; 8 Department of Medicine, Robert H. Lurie Comprehensive Cancer Center, Northwestern University Feinberg School of Medicine, Chicago, IL, USA; 9 Department of Medicine, Mayo Clinic, Jacksonville, FL, USA; 10 The Angeles Clinic and Research Institute, Santa Monica, CA, USA; 11 Department of Medical Oncology, Hofstra North Shore-LIJ School of Medicine, ProHEALTH Care Associates, Lake Success, NY, USA; 12 Department of Medicine, Memorial Sloan-Kettering Cancer Center, New York, NY, USA; 13 Department of Medicine, Dana Farber Cancer Institute, Harvard Medical School, Boston, MA, USA; 14 Department of Medicine, Lineberger Comprehensive Cancer Center, University of North Carolina, Chapel Hill, NC, USA; 15 Department of Pathology, Huntsman Cancer Center, University of Utah, Salt Lake City, UT, USA; 16 Department of Pathology and Laboratory Medicine, University of British Columbia, Vancouver, BC, USA; 17 Department of Genetics, Lineberger Comprehensive Cancer Center, University of North Carolina, Chapel Hill, NC, USA; 18 Department of Medical Oncology, Siteman Cancer Center, Washington University School of Medicine, St. Louis, MO, USA; 19 Department of Biostatistics and Computational Biology, Alliance Statistics and Data Center, Dana Farber Cancer Institute, Boston, MA, USA

## Abstract

PAM50 intrinsic breast cancer subtypes are prognostic independent of standard clinicopathologic factors. CALGB 9741 demonstrated improved recurrence-free (RFS) and overall survival (OS) with 2-weekly dose-dense (DD) versus 3-weekly therapy. A significant interaction between intrinsic subtypes and DD-therapy benefit was hypothesized. Suitable tumor samples were available from 1,471 (73%) of 2,005 subjects. Multiplexed gene-expression profiling generated the PAM50 subtype call, proliferation score, and risk of recurrence score (ROR-PT) for the evaluable subset of 1,311 treated patients. The interaction between DD-therapy benefit and intrinsic subtype was tested in a Cox proportional hazards model using two-sided alpha=0.05. Additional multivariable Cox models evaluated the proliferation and ROR-PT scores as continuous measures with selected clinical covariates. Improved outcomes for DD therapy in the evaluable subset mirrored results from the complete data set (RFS; hazard ratio=1.20; 95% confidence interval=0.99–1.44) with 12.3-year median follow-up. Intrinsic subtypes were prognostic of RFS (*P*<0.0001) irrespective of treatment assignment. No subtype-specific treatment effect on RFS was identified (interaction *P*=0.44). Proliferation and ROR-PT scores were prognostic for RFS (both *P*<0.0001), but no association with treatment benefit was seen (*P*=0.14 and 0.59, respectively). Results were similar for OS. The prognostic value of PAM50 intrinsic subtype was greater than estrogen receptor/HER2 immunohistochemistry classification. PAM50 gene signatures were highly prognostic but did not predict for improved outcomes with DD anthracycline- and taxane-based therapy. Clinical validation studies will assess the ability of PAM50 and other gene signatures to stratify patients and individualize treatment based on expected risks of distant recurrence.

## Introduction

The clinical and genomic heterogeneity of early-stage breast cancer is well-recognized. Tumor characterization beyond hormone receptor status, HER2 status, tumor size, and extent of nodal involvement may improve prognostication and guide systemic therapy. Intrinsic breast cancer subtypes derived through global gene-expression analysis are prognostic independent of standard clinicopathological variables and identify the subgroup(s) of patients most likely to benefit from a given adjuvant chemotherapy regimen.^1–5^

The luminal A (LumA), luminal B (LumB), HER2-enriched (HER2-E), basal-like, and normal-like breast cancer subtypes were initially defined through unsupervised clustering analysis of global gene expression from RNA extracted from frozen tissue.^1^ A 50-gene qPCR assay (PAM50) was developed to identify the intrinsic biological subtypes using RNA isolated from more readily available formalin-fixed, paraffin-embedded (FFPE) tissue. These subtypes can also be assessed using a multiplexed gene-expression profiling technology (NanoString Technologies; Seattle, WA, USA). The PAM50 assay was used to develop a prognostic risk of relapse score based on the relative distance to the centroid of each subtype;^6^ a proliferation score based on a subset of genes related to cell cycle progression;^7^ and composite scores that include tumor size with molecular phenotypes.^6,7^ Although each has prognostic capability, the utility of these scores to predict for specific treatment benefit and select therapy has not been studied.

The CALGB (Alliance) 9741 adjuvant node-positive breast cancer trial randomized treatment with doxorubicin (A), cyclophosphamide (C), and paclitaxel (T) using a 2×2 factorial design. The two factors were (i) 2-weekly (dose dense; DD) versus 3-weekly administration and (ii) sequential (A→T→C) versus concurrent (AC→T) chemotherapy. DD therapy improved recurrence-free survival (RFS) and overall survival (OS).^8^ No survival differences were observed between concurrent and sequential administration, and no interaction between density and sequence was identified. An unplanned retrospective subset analysis suggested an interaction between estrogen receptor (ER) status and DD-therapy benefit.^9^ We hypothesized that the increased prognostic accuracy of PAM50 would allow for the prediction of benefit with DD scheduling.

## Results

### Patient and tumor characteristics

PAM50 intrinsic subtype calls were generated for 1,321 of 1,471 patients (90%) with evaluable blocks or slides. There was a slight, but statistically significant, enrichment of ER-negative and progresterone receptor-negative cancers (both *P*<0.05) in the PAM50 sample set relative to the treated study population (*N*=1,972). On average, tumor size was larger in the PAM50 subset (*P*<0.001), as expected with considerations for sample acquisition and processing. Treatment assignment and other patient characteristics remain well balanced in the evaluable population (Table 1).

At 12.3 years median follow-up in all treated patients, 664 recurrences or deaths have been recorded in C9741, 452 of which occur in the subset evaluable by PAM50. With updated outcomes information, the overall treatment effects are consistent with the primary C9741 clinical trial results.^8^ No differences in RFS or OS were observed between sequential versus concurrent chemotherapy: hazard ratio (HR)=1.06 (95% confidence interval (CI)=0.91–1.24, *P*=0.43) and HR=1.04 (95% CI=0.89–1.23, *P*=0.63), respectively. Improved outcomes were seen in patients who received DD treatment: HR=1.26 for RFS (95% CI=1.08–1.47, *P*=0.003) and HR=1.21 for OS (95% CI=1.03–1.43, *P*=0.019). The effect of dose density on RFS and OS is slightly attenuated in the PAM50 subset (HR=1.20 (95% CI=0.99–1.44) and HR=1.15 (95% CI=0.95–1.40), respectively) and with the smaller sample size does not reach statistical significance at the 0.05 level.

### Distribution of PAM50 subtypes and prognostic value for RFS and OS

PAM50 generated 414 (32%) LumA, 338 (26%) LumB, 266 (20%) HER2-E, and 293 (22%) basal-like calls. Patient characteristics were broadly distributed across intrinsic subtypes (Supplementary Table 1). The relationship between PAM50 intrinsic subtype and clinical prognostic factors was consistent with previous reports, including the enrichment of LumA cancers in postmenopausal patients and among smaller tumors. Randomized treatment assignment was well balanced across subtypes.

**Figure 1:**
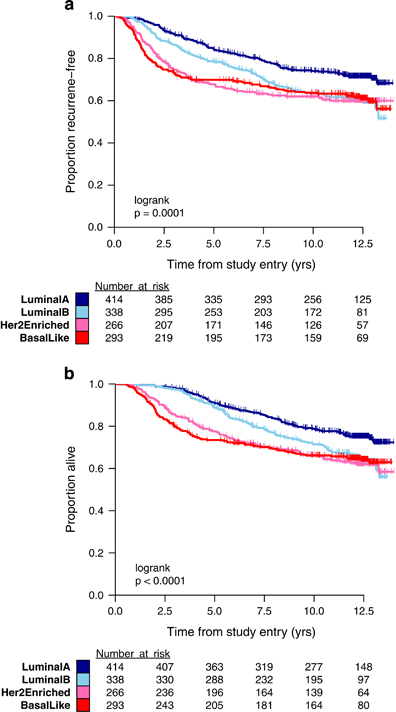
(**a**) Kaplan–Meier plot of RFS in C9741 patients classified by PAM50 intrinsic subtype: basal-like, HER2-E, LumA, and LumB. (**b**) Kaplan–Meier plot of OS in C9741 patients classified by PAM50 intrinsic subtype: basal-like, HER2-E, LumA, and LumB. OS, overall survival; RFS, recurrence-free survival.

The prognostic relationship between intrinsic subtype and RFS was statistically significant (logrank *P*=0.0001) and demonstrated patterns consistent with previous studies (Figure 1a).^6^ A higher rate of recurrence was observed with LumB (HR=1.50, 95% CI=1.16–1.93), HER2-E (HR=1.70, 95% CI=1.30–2.22), and basal-like (HR=1.66, 95% CI=1.28–2.16) tumors relative to LumA tumors. Furthermore, basal-like and HER2-E subtypes have substantially higher rates of recurrence than LumA and LumB in years 0–3 and lower rates of recurrence afterwards. The independent prognostic value of intrinsic subtype after adjusting for the number of positive nodes and menopausal status is summarized in Table 2. A similar relationship between intrinsic subtype and OS is demonstrated (Figure 1b).

### PAM50 intrinsic subtype does not predict benefit with DD therapy

**Figure 2:**
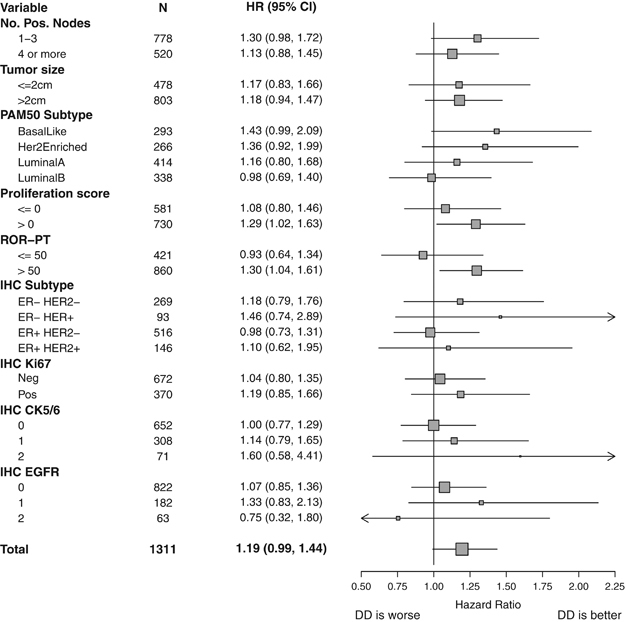
Forest plot displaying hazard ratios (HR) and 95% confidence intervals (CI) for RFS with DD therapy in patient subgroups from C9741 defined by tumor characteristics (number of positive nodes and tumor size), PAM50 assay (intrinsic subtype, proliferation score, and ROR-PT score), and immunohistochemistry (ER/HER2, Ki67, CK5/6, and EGFR). CK, cytokeratin; DD, dose dense; EGFR, epidermal growth factor receptor; ER, estrogen receptor; IHC, immunohistochemistry; N, number of subjects; RFS, recurrence-free survival; ROR-PT, risk of recurrence score.

The ability of PAM50 subtype to predict for benefit with adjuvant DD chemotherapy was evaluated as a test of interaction between dose density and the four subtype calls. No statistically significant association with RFS (3 df, *P*=0.44) or OS benefit (3 df, *P*=0.65) was identified. As an exploratory analysis, levels of RFS benefit from DD treatment were evaluated within patient subsets defined by PAM50 and patient/tumor characteristics (Figure 2). The forest plot suggests that the benefit of dose density was most substantial in the basal-like and HER2-E subtypes. A larger study is required to confirm this effect.

### PAM50 proliferation and ROR-PT scores: prognostic and predictive value

**Figure 3:**
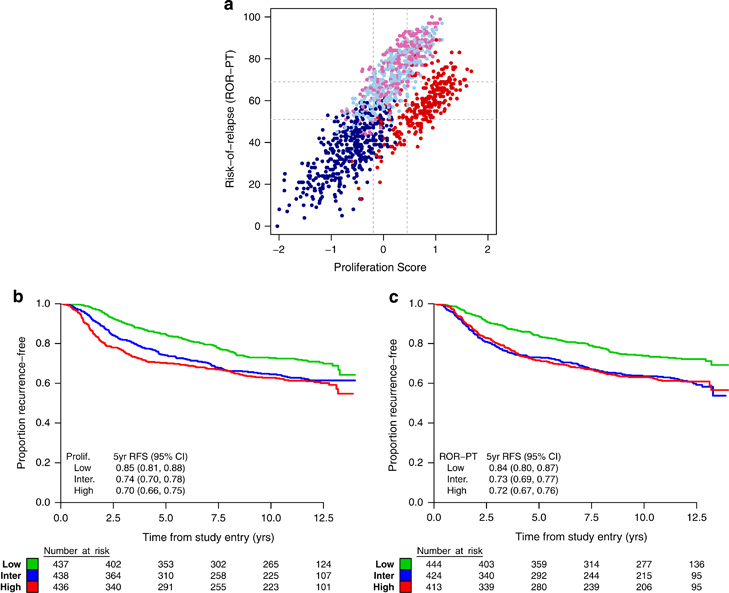
(**a**) Scatterplot of proliferation and ROR-PT scores labeled by intrinsic subtype: basal-like (red), HER2-E (pink), LumA (dark blue), and LumB (light blue). Cutpoints that divide each score into tertiles are shown in gray. (**b**) Kaplan–Meier plot and 5-year RFS estimates for the low, intermediate (inter.), and high subgroups of proliferation (prolif.) scores. (**c**) Kaplan–Meier plot and 5-year RFS estimates for the low, intermediate, and high subgroups of ROR-PT scores. CI, confidence interval; RFS, recurrence-free survival; ROR-PT, risk of recurrence score.

Proliferation score and ROR-PT score were considered as continuous variables in all inferential tests because the thresholds for classification (i.e., cutoff values for high/intermediate/low risk) had not been established in this patient population. A strong positive correlation was observed between proliferation score and ROR-PT score (*r*=0.72), and each is associated with intrinsic subtype as expected from the shared genomic features to each algorithm (Figure 3a).

Proliferation and ROR-PT scores were strongly prognostic for RFS in C9741 when evaluated as linear terms in the Cox proportional hazard model. For proliferation score, a 0.5-unit change corresponded to an 18% increase in risk of recurrence (HR=1.17, 95% CI=1.10–1.26, *P*<0.0001, Figure 3b). For ROR-PT score, a 10-unit change corresponded to a 12% increase in risk of recurrence (HR=1.12, 95% CI=1.07–1.18, *P*<0.0001, Figure 3c). Menopausal status did not affect the prognostic value of these scores (Supplementary Figure 2). Conversely, no statistically significant associations with DD therapy were seen using interaction tests in the bivariable Cox models (1 df, *P*=0.14 and 0.58, respectively). Similar prognostic relationships between RFS and intrinsic subtype, proliferation score, and ROR-PT score were found in HER2-negative patients as a planned subset analysis (*N*=848; data not shown). No interaction between dose density and RFS was observed, driven partly by the lack of overall benefit observed in this patient subgroup (HR=1.03 for RFS, 95% CI=0.82–1.30, *P*=0.7958).

The hazard of breast cancer recurrence is known to change over time by intrinsic subtype^10^ and proliferation,^11^ and this was observed in C9741 (Grambsch and Therneau, *P*<0.0001 for each). Therefore, we performed a sensitivity analysis of the predictive value of the PAM50 assay for early recurrence by 3 years and all recurrences by 10 years. The relative benefit of DD therapy for early and late recurrence was explored across each PAM50 molecular score using nonparametric spline regression models (Supplementary Figure 3). The significant increases in overall risk of recurrence are seen most strongly in the lower ranges of proliferation and ROR-PT scores, whereas nonsignificant trends of DD-therapy benefit are seen only with higher scores. The Kaplan–Meier estimates and hazard ratios of DD therapy are displayed in Figures 2 and 3 using cut-points that give approximately equally sized tertiles. The greatest prognostic difference is between low versus intermediate/high, whereas the nonsignificant trends of predicting DD-therapy benefit occur only in patients with intermediate/high scores (Supplementary Figures 3A and D). Determinations of optimal thresholds and statistical significance will require validation in independent data sets and were not performed as part of this exploratory analysis.

### Comparison of PAM50 phenotypes to immunohistochemistry assessments of ER/HER2, Ki67, CK5/6, epidermal growth factor receptor

Substantial agreement was seen between site-determined and centralized assessments of ER by tissue microarray (Cohen’s *Κ*=0.78). Common relationships between intrinsic subtypes are noted for the 1,024 cases with both PAM50 subtype call and ER/HER2 immunohistochemistry (IHC) results (Table 3). LumA and LumB tumors were predominantly ER-positive. Basal-like tumors were predominantly ER-negative. The distribution of intrinsic subtypes did not vary by HER2 IHC staining when stratified by ER status (Mantel–Haenszel *χ* ^2^, *P*=0.43). Ki67-positive tumors were highly enriched in basal-like and to a lesser degree in HER2-E subtypes relative to the luminal subtypes (Pearson’s *χ* ^2^, *P*<0.0001). Similar patterns were seen for cytokeratin (CK) 5/6 and epidermal growth factor receptor (EGFR) 1+/2+ tumors (mean score *χ* ^2^, *P*<0.0001).

When considering breast cancer subtypes by PAM50 and clinicopathologic variables using the multivariable Cox models in Table 2, the prognostic value of PAM50 intrinsic subtype remained statistically significant in a model including subtype by both assessments (*P*=0.004). Conversely, RFS did not vary significantly by IHC subtype defined by ER/HER2 alone (*P*=0.31) or in conjunction with Ki67, CK5/6, and EGFR (*P*=0.12). Thus, the cumulative prognostic value of PAM50 and ER/HER2 by IHC is largely captured by intrinsic subtype alone in this cohort of patients (data not shown).

## DISCUSSION

Precision medicine in oncology has been spurred in part by the availability of multigene-based mRNA expression assays intended to add prognostic and predictive value to traditional markers of risk (e.g., tumor size, nodal status). For breast cancer, the identification of several molecular subgroups with distinct clinical outcomes is possible through commercial assays.^12–15^ Because of similar prognostic performance, it is likely these signatures are derived from similar biologic principles. Unfortunately, none reliably predict for benefit with specific chemotherapeutics, including the addition of taxanes. Practically speaking, there is great need for a single platform that can be applied to all breast primaries, performed on small amounts of routinely processed tissue (e.g., FFPE), assessed in local laboratories, and easily interpreted for general clinical use.

High quality tumor samples from a large, representative subset of participants in C9741 were available for this study. PAM50 was assessed with the same nanotechnology-based nCounter digital gene-expression platform as the Prosigna Breast Cancer Prognostic Gene Signature Assay. All subtypes were represented in a distribution similar to that of other populations unselected for hormone receptor or HER2 status.^6^ Our findings confirm the prognostic value of the PAM50 intrinsic subtype identified in smaller studies of patients treated with contemporary adjuvant anthracycline and taxane-based regimens, including GEICAM/9906.^16^ Intrinsic subtype, proliferation score, and ROR-PT score were strongly associated with RFS and OS irrespective of treatment assignment and independent of standard clinicopathologic variables. Comparison of subtypes defined by PAM50 or IHC demonstrates that prognosis is most reliably determined by intrinsic subtype as opposed to conventional assessments of ER/HER2 status.

PAM50 testing also identified patterns of recurrence. Higher rates of recurrence were observed with the non-luminal versus luminal subtypes between years 0 and 3, but lower rates of recurrence were observed thereafter. This is consistent with clinical observations of late recurrences in endocrine responsive breast cancer and early recurrences in the poor prognosis subset of triple-negative and untreated HER2-positive disease, suggesting that intrinsic subtyping is more precisely informative.

The updated survival benefits associated with DD AC→T remain unchanged.^8,17^ The strong prognostic differences by PAM50 intrinsic subtype, proliferation score, and ROR-PT score were seen regardless of treatment assignment, but no test was specifically predictive of subgroups with greater or lesser benefit from DD scheduling. As expected, a suggestion of greatest benefit was observed in the chemotherapy sensitive (i.e., basal-like and HER2-E) subtypes and with higher ROR-PT scores and proliferation. Therefore, one cannot definitively conclude that PAM50 does not predict for DD-therapy benefit, as this study may be underpowered to detect such an interaction. (Neo)adjuvant chemotherapy benefit in the higher PAM50 recurrence risk subtypes has been previously reported.^6,18,19^ Available data are concordant in that the LumA subtype is associated with a more favorable natural history and greater sensitivity to endocrine therapy, whereas the basal-like and HER2-E subtypes are associated with poorer clinical outcomes and greater sensitivity to chemotherapy. In contrast, the prognosis at 10 years for the LumB group is as poor as the HER2-E and basal-like groups.

Intrinsic subtyping was prognostic in this study, but improved survival with DD treatment in patient subgroups defined by intrinsic subtype did not reach statistical significance in a full interaction model. This may be attributed to a lack of power in the smaller evaluable subset of patients treated in C9741, and to the strong prognostic differences between LumA and LumB versus the basal-like and HER-E subtypes. The prediction of treatment benefit remains a key goal, and clinical validation studies will further assess the ability of the PAM50 gene signature to stratify patients on the risk of distant recurrence and maximize the reliable identification of patients (i.e., the LumA population) with such favorable long-term outcomes that they should be spared unnecessary adjuvant chemotherapy.

## MATERIALS AND METHODS

### Patient enrollment, sample acquisition, clinical outcome

Cancer and Leukemia Group B (CALGB) 9741 was conducted in collaboration with the Eastern Cooperative Group, Southwest Oncology Group, and North Central Cancer Treatment Group, accruing 2,005 subjects between September 1997 and March 1999.^8^ Clinical endpoints included OS and RFS, defined as the interval from study entry until first local or distant recurrence or death owing to any cause.^20^ Survival analyses are based on updated clinical outcomes data collected through January 2012.

A total of 1,652 patients had FFPE primary breast tumor samples archived at the CALGB Pathology Coordinating Office (PCO), of which 1,471 were suitable for inclusion in this study. Gene-expression profiles were generated for 1,321 of 1,471 patient samples (90%). Ten randomized subjects did not receive treatment and were excluded. The primary analysis therefore includes 1,311 patients in total (REMARK diagram,^21^ Supplementary Figure 1).

### Sample preparation and multiplexed gene-expression profiling

The CALGB PCO provided batches of 96 tumor samples as block punches or slide material. To avoid technical batch effects, all available high, moderate, and poor slide materials were randomly assigned to batches by the CALGB (Alliance) Statistical Center using permuted-blocks. FFPE samples were sent to Washington University CLIA molecular laboratories for macrodissection of slide material (if needed) and RNA extraction using an RNA isolation kit and procedures provided by NanoString Technologies. Optical density of total RNA was measured at 260 and 280 nm to determine yield and purity using a low-volume spectrophotometer. RNA samples passed quality control if the measured concentration was ⩾12.5 ng/μl and the A260/280 ratio was 1.7–2.5. A second optical density measurement was taken for RNA samples that failed to meet the quality metrics before exclusion. Gene-expression profiling was performed on a research-use-only nCounter Analysis System using the research-use-only PAM50 probe set. The hybridization reaction was performed according to procedures provided by NanoString Technologies using a nominal RNA input of 250 ng. The hybridization time was 15–21 h using a bench-top thermocycler set to 65 °C with a heated lid set to 70 °C. Manufacturer's specifications were used for the nCounter Prep Station, which prepares the hybridized products for imaging. The nCounter Digital Analyzer reports the digital counts representing the number of molecules labeled with a fluorescent barcode for each probe-targeted transcript. The Digital Analyzer was set to scan at the ‘max’ sensitivity setting defined as 1155 FOV (fields of view).

Raw gene-expression data (RCC files) were evaluated using pre-specified quality metrics and have been deposited in the Gene Expression Omnibus (GSE74821). The geometric mean of eight housekeeping genes was required to be above a minimum threshold to ensure gene-expression signal levels sufficient for accurate and precise results. Data that passed sample and assay quality metrics were provided in a blinded fashion to NanoString Technologies for normalization and analysis with a proprietary PAM50 algorithm.^22^ Gene-expression profiles were returned as a four-level classifier (LumA, LumB, basal-like, and HER2-E) based upon Pearson’s distance to centroids re-trained on the nCounter platform; a proliferation score that represents an average of expression values for the subset of proliferation-related genes (expanded from 11 in the original model to 18 in the NanoString version);^7^ and a ROR-PT score expanded from the original risk of relapse model.^6^ The NanoString ROR-PT algorithm includes distance to all centroids, proliferation score, and gross pathologic tumor size as terms to the model. ROR-PT scores were calculated by NanoString Technologies assuming that samples were from small (⩽2 cm) or large (>2 cm) tumors to maintain the blind-to-patient information.

### Immunohistochemical analyses

Centralized whole-section analysis results for HER2 were available from 1,224 of the 1,652 C9741 patients with submitted FFPE primary breast tumor samples. HER2 staining was performed with the CB11 monoclonal antibody (BioGenex Laboratories, San Ramon, CA, USA; #MU134-UC). Cases were considered positive with staining of ⩾50% carcinoma cells.^23,24^

Blocks suitable for inclusion on a tissue microarray were obtained from 1,231 C9741 patients and reviewed to identify representative areas of viable invasive breast carcinoma. Replicate 0.6 mm cores from each case were extracted and assembled into separate tissue microarrays at the CALGB PCO using established methods.^25^ Duplicate blocks (i.e., two cores per patient) were used. A total of 26 sections (4 microns each) were cut and shipped to the Genetic Pathology Evaluation Centre, British Columbia Cancer Agency (Vancouver, BC, Canada) or the University of Colorado, School of Medicine (Denver, CO, USA). Guidelines for IHC-staining conditions and interpretation of the ER (LabVision, Fremont, CA, USA; #RM-9101), Ki67 (LabVision, #RM-9106), CK5/6 (Zymed Laboratories, San Francisco, CA, USA; Clone D5/16B4, #18–0267), and EGFR (Dako Corporation, Carpinteria, CA, USA; #K1492) assays were pre-specified.^26,27^ Staining was performed within 1 week of tissue microarray sectioning, and all biomarker scoring was performed by pathologists blinded to patient data. For continuously quantified variables (ER, Ki67), the average between replicate cores was used. For semiquantitative variables (CK5/6, EGFR), the higher score was taken. Centralized IHC staining of ER and HER2 was available on 1,124 C9741 patients, including 1,024 of the cases successfully profiled for PAM50.

### Statistical analysis

Descriptive statistics were used to summarize clinical and molecular endpoints. Contrasts of demographics and tumor characteristics between patient subgroups were evaluated using Pearson’s *χ* ^2^ test with continuity correction for categorical variables, and Wilcoxon rank-sum tests for continuous variables. Survival functions for time-to-event endpoints and median follow-up were summarized using the Kaplan–Meier product limit estimator. HRs and CIs were estimated using univariable and multivariable Cox proportion hazards models. Planned prospective analyses of the interaction between dose density and PAM50 intrinsic subtype (categorical), proliferation score (continuous), and ROR-PT score (continuous) were performed using score tests for bivariable Cox proportional hazard models. Planned comparisons of molecular phenotypes by PAM50 and IHC were performed using likelihood ratio tests for nested multivariable Cox models. Correlation between molecular phenotypes was evaluated using Pearson’s *χ* ^2^ tests for binary covariates and Mantel–Haenszel *χ* ^2^ tests for ordinal covariates and stratified models. All the tests used a two-sided type I error of alpha=0.05. Exploratory analyses of molecular phenotypes were performed using nonlinear knotted cubic spline function (knots at evenly spaced quintiles) and logistic regression models for 3- and 10-year rates of recurrence.^28^

All statistical analyses were performed using SAS v9.2 (Cary, NC, USA). Graphics were generated in R version 2.15.0.^29^

## Figures and Tables

**Table 1:** Patient and tumor characteristics

*Variable*	*All treated C9741 patients (*N=*1,972)*^a^	*Subset evaluable by PAM50 (*N=*1,311)*^b^	P-*value*^c^
Number of positive nodes; median (IQR)	3 (1, 5)	3 (1, 5)	0.655
Age in years; median (IQR)	50 (43, 57)	50 (43, 57)	0.697

*Tumor size*			
⩽2 cm	787 (40%)	478 (36%)	<0.001
>2 cm	1140 (58%)	803 (61%)	
Missing	45 (2%)	30 (2%)	

*ER status*			
Positive	1275 (65%)	822 (63%)	0.022
Negative	663 (33%)	468 (36%)	
Missing	34 (2%)	21 (1%)	

*PgR status*			
Positive	1108 (56%)	706 (54%)	0.008
Negative	821 (42%)	578 (44%)	
Missing	43 (2%)	27 (2%)	

*Menopausal status*			
Pre	976 (49%)	642 (49%)	0.513
Post	996 (51%)	669 (51%)	

*Treatment arm*			
Sequential—q3	483 (25%)	314 (24%)	0.408
Sequential—q2	493 (25%)	343 (26%)	
Concurrent—q3	501 (25%)	330 (25%)	
Concurrent—q2	495 (25%)	324 (25%)	

*Recurrence-free survival at*			
3 years (95% CI)	0.84 (0.82, 0.85)	0.83 (0.81, 0.85)	0.402
5 years (95% CI)	0.77 (0.75, 0.79)	0.76 (0.74, 0.79)	
10 years (95% CI)	0.67 (0.65, 0.69)	0.67 (0.64, 0.69)	

*Overall survival at*			
3 years (95% CI)	0.92 (0.90, 0.93)	0.91 (0.90, 0.93)	0.403
5 years (95% CI)	0.85 (0.83, 0.86)	0.84 (0.82, 0.86)	
10 years (95% CI)	0.72 (0.70, 0.74)	0.72 (0.69, 0.74)	

Abbreviations: CI, confidence interval; ER, estrogen receptor; IQR, interquartile range.

a *N*=1,973 patients were reported in the primary manuscript, but one patient was later excluded having never begun treatment.

b *N*=1,311 because 10 patients with PAM50 genomic results never started protocol directed therapy.

c *P*-values are for comparisons of the 1,311 patients evaluable for PAM50 versus the 661 treated patients who were not evaluable. Comparisons for categorical variables use Pearson's *χ*^2^ test; for continuous variables use Wilcoxon rank-sum tests; and for time-to-event variables use logrank tests.

**Table 2:** Multivariable Cox proportional hazard models of RFS and OS

*Variable (contrast)*	*Recurrence-free survival*		*Overall survival*	
	*Hazard ratio*	P*-value*	*Hazard ratio*	P*-value*
Number of positive nodes (sqrt)	2.17 (1.82, 2.60)	<0.0001	2.18 (1.82, 2.63)	<0.0001
Menopausal status (pre/post)	0.91 (0.75, 1.10)	0.3276	0.88 (0.72, 1.08)	0.2284
Dose density (q3wk/q2wk)	1.20 (0.99, 1.45)	0.0582	1.15 (0.94, 1.40)	0.1671
Sequence of therapy (con/seq)	0.98 (0.81, 1.18)	0.8314	0.98 (0.80, 1.19)	0.8174

*PAM50 intrinsic subtype*				
Basal-like versus LumA	1.83 (1.40, 2.38)	<0.0001	1.91 (1.44, 2.53)	<0.0001
HER2-E versus LumA	1.63 (1.24, 2.15)		1.69 (1.27, 2.26)	
LumB versus LumA	1.47 (1.14, 1.91)		1.47 (1.12, 1.94)	

Abbreviations: con, concurrent; HER2-E, HER2-enriched; Lum, luminal; q2wk, every two weeks or 2-weekly; q3wk, every 3 weeks or 3-weekly; seq, sequential; sqrt, square root.

*N*=1,299 because 11 patients were missing information about the number of positive nodes or menopausal status.

**Table 3:** Prevalence of molecular phenotypes by IHC and PAM50 intrinsic subtype

*Variable*	*Basal-like (*N=*293)*	*HER2-E (*N=*266)*	*LumA (*N=*414)*	*LumB (*N=*338)*
*IHC subtype*^a^				
Unknown	64	53	118	52
ER− HER2−	161 (70%)	85 (40%)	15 (5%)	8 (3%)
ER− HER2+	50 (22%)	27 (13%)	11 (4%)	5 (2%)
ER+ HER2−	14 (6%)	80 (38%)	208 (70%)	214 (75%)
ER+ HER2+	4 (2%)	21 (10%)	62 (21%)	59 (21%)

*Ki67*				
Unknown	60	54	111	44
Neg. (<13.5%)	67 (29%)	112 (53%)	291 (96%)	202 (69%)
Pos. (⩾13.5%)	166 (71%)	100 (47%)	12 (4%)	92 (31%)

*CK5/6*				
Unknown	61	52	121	47
0	75 (32%)	121 (57%)	225 (77%)	231 (79%)
1	99 (43%)	88 (41%)	61 (21%)	60 (21%)
2	58 (25%)	5 (2%)	7 (2%)	1 (0%)

*EGFR*				
Unknown	56	48	101	39
0	76 (32%)	161 (74%)	297 (95%)	288 (96%)
1	118 (50%)	41 (19%)	13 (4%)	10 (3%)
2	43 (18%)	16 (7%)	3 (1%)	1 (0%)

Abbreviations: CK, cytokeratin; EGFR, epidermal growth factor receptor; HER2-E, HER2-enriched; IHC, immunohistochemistry; Lum, luminal; Neg., negative; Pos., positive.

Column percentages are computed excluding samples with unknown status by IHC.

a ER-positivity is defined by ≥1% positive tumor nuclei. HER2-positivity is defined by staining of >50% carcinoma cells.
