# Supplementary material for: PAM50 gene signatures and breast cancer prognosis with adjuvant anthracycline- and taxane-based chemotherapy: correlative analysis of C9741 (Alliance)
Source: NPJ Breast Cancer. 2016 Jan 6;2:15023–. doi: 10.1038/npjbcancer.2015.23 (PMC5501351; doi:10.1038/npjbcancer.2015.23)

**Supplemental Table 1: Patient and tumor characteristics by PAM50 intrinsic subtype.**

| **Variable** | **Basal-like**  **(N=293)** | **HER2-E**  **(N=266)** | **LumA**  **(N=414)** | **LumB**  **(N=338)** | **p-value^2^** |
| --- | --- | --- | --- | --- | --- |
| Number of positive nodes; median (IQR) | 2 (1, 4) | 3 (1, 6) | 3 (1, 5) | 3 (1, 5) | 0.0202 |
| Age in years; median (IQR) | 47 (41, 54) | 49 (42, 57) | 51 (46, 58) | 50 (42, 58) | < 0.0001 |
| Tumor size  ≤ 2 cm  > 2 cm  Unknown | 90 (31%)  203 (69%)  0 (0%) | 87 (33%)  171 (64%)  8 (3%) | 191 (46%)  210 (51%)  13 (3%) | 110 (33%)  219 (65%)  9 (3%) | < 0.0001 |
| ER status  Positive  Negative  Unknown | 27 (9%)  265 (90%)  1 (<1%) | 118 (44%)  143 (54%)  5 (2%) | 363 (88%)  41 (10%)  10 (2%) | 314 (93%)  19 (6%)  5 (1%) | < 0.0001 |
| PgR status  Positive  Negative  Unknown | 26 (9%)  266 (91%)  1 (<1%) | 98 (37%)  161 (61%)  7 (3%) | 319 (77%)  84 (20%)  11 (3%) | 263 (78%)  67 (20%)  8 (2%) | < 0.0001 |
| HER2 status  Positive  Negative  Unknown | 60 (20%)  188 (64%)  45 (15%) | 48 (18%)  175 (66%)  43 (16%) | 81 (20%)  249 (60%)  84 (20%) | 67 (20%)  236 (70%)  35 (10%) | 0.7951 |
| Menopausal status  Pre  Post | 161 (55%)  132 (45%) | 141 (53%)  125 (47%) | 171 (41%)  243 (59%) | 169 (50%)  169 (50%) | 0.0012 |
| Treatment arm  Sequential – q3  Sequential – q2  Concurrent – q3  Concurrent – q2 | 68 (23%)  80 (27%)  74 (25%)  71 (24%) | 57 (21%)  68 26%)  65 (24%)  76 (29%) | 106 (26%)  94 (23%)  102 (25%)  112 (27%) | 83 (25%)  101 (30%)  89 (26%)  65 (19%) | 0.2076 |
| Recurrence-free survival at  3 years (95% CI)  5 years (95% CI)  10 years (95% CI) | 0.73 (0.68, 0.78)  0.70 (0.64, 0.75)  0.64 (0.58, 0.69) | 0.75 (0.69, 0.80)  0.68 (0.62, 0.73)  0.62 (0.56, 0.68) | 0.92 (0.89, 0.94)  0.85 (0.81, 0.88)  0.74 (0.69, 0.78) | 0.87 (0.82, 0.90)  0.79 (0.74, 0.83)  0.64 (0.58, 0.69) | < 0.0001 |
| Overall survival at  3 years (95% CI)  5 years (95% CI)  10 years (95% CI) | 0.80 (0.75, 0.84)  0.73 (0.68, 0.78)  0.66 (0.60, 0.71) | 0.86 (0.81, 0.89)  0.78 (0.72, 0.82)  0.66 (0.60, 0.72) | 0.98 (0.96, 0.99)  0.91 (0.88, 0.93)  0.79 (0.74, 0.83) | 0.97 (0.95, 0.99)  0.89 (0.85, 0.92)  0.71 (0.66, 0.76) | < 0.0001 |
| ^1^Percentages are calculated as column percentages.  ^2^Kruskal-Wallis test for continuous variables, Chi-Square Test for categorical variables, Log-Rank test for time to event variables. | | | | | |

**FIGURE LEGENDS**

**Supplemental Figure 1.** REMARK diagram for the evaluation of PAM50 in C9741 indicating (i) the total number of patients enrolled and randomized, (ii) the number of biospecimens collected and allocated for mRNA expression assays, (iii) the total number of successful mRNA expression results, (iv) the final analysis cohort for PAM50, and (v) the subset with immunohistochemistry for ER and HER2.

**Supplemental Figure 2.** Kaplan-Meier plots for the Low, Intermediate, and High tertiles of ROR-PT and Proliferation scores within subgroups of premenopausal and postmenopausal patients. Tests of interaction of tertiles with menopausal status in bivariable Cox proportional hazard models failed to reach statistical significance (p=0.78 and p=0.22 for ROR-PT and Proliferation scores, respectively).

**Supplemental Figure 3.** Exploratory analyses of the relationship between DD treatment (Q2 versus Q3 regimen) and molecular phenotypes. Kaplan Meier plots of treatment arm by tertiles of ROR-PT and Proliferation Score are shown in A and D, respectively. The risks of recurrence at 3 and 10 years were fit to non-linear logistic regression models in panels B-C and E-F and displayed with 95% confidence bands. Models contain an interaction between dose density and knotted cubic spline function for ROR-PT or Proliferation Score. In all models, non-linear associations of molecular phenotype to risk of recurrence are statistically significant, but the interactions with dose density are non-significant.

**Supplemental Figure 1**


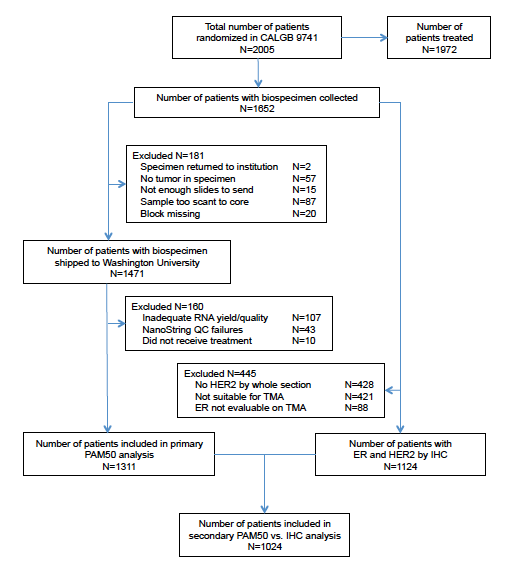


**Supplemental Figure 2**

**Supplemental Figure 3**


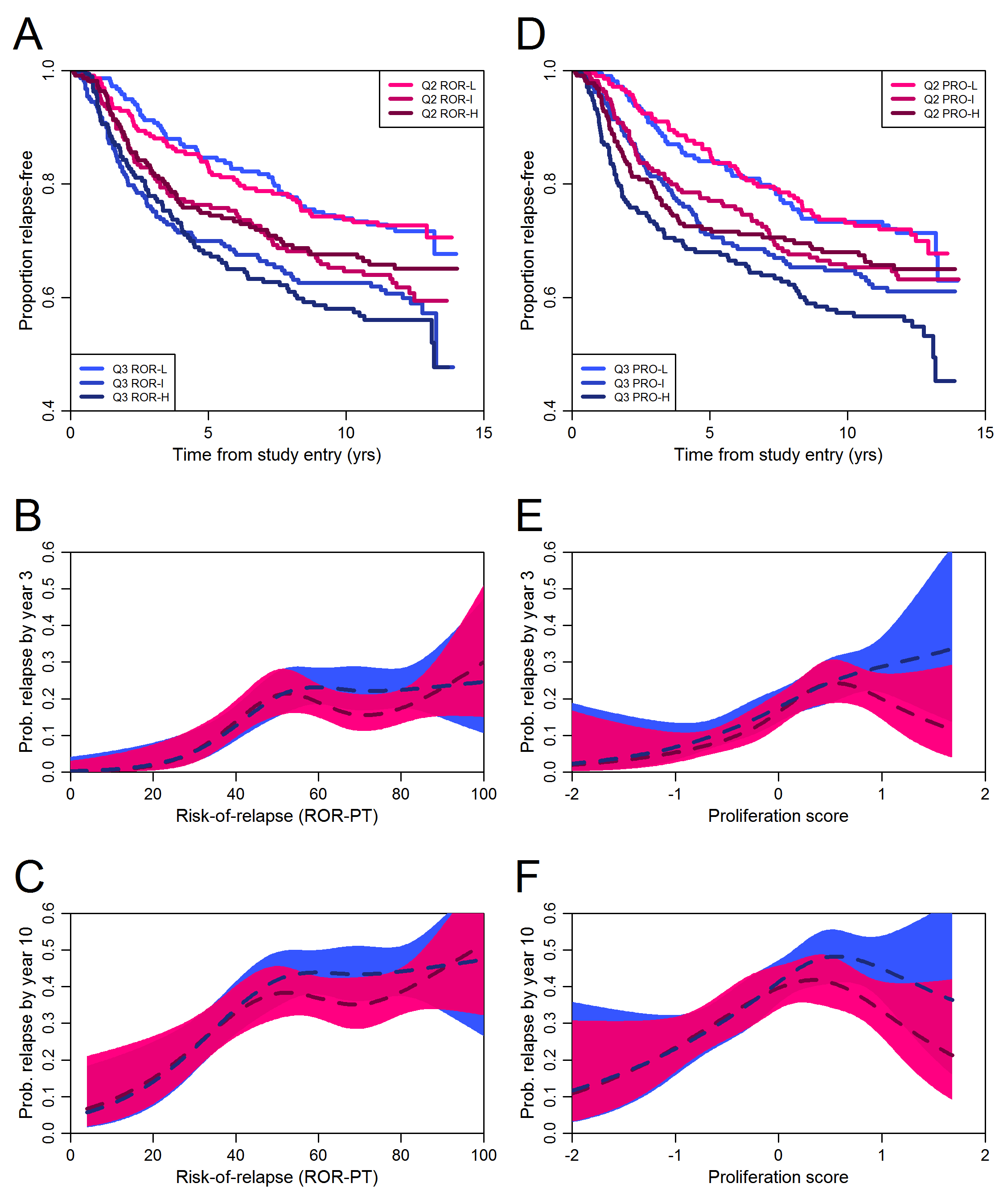

Supplement: Supplemental Information [file npjbcancer201523-s1.doc]
